# Combination of Prehospital NT-proBNP with qSOFA and NEWS to Predict Sepsis and Sepsis-Related Mortality

**DOI:** 10.1155/2022/5351137

**Published:** 2022-02-23

**Authors:** Francisco Martín-Rodríguez, Laura Melero-Guijarro, Guillermo J. Ortega, Ancor Sanz-García, Teresa de la Torre de Dios, Jesús Álvarez Manzanares, José L. Martín-Conty, Miguel A. Castro Villamor, Juan F. Delgado Benito, Raúl López-Izquierdo

**Affiliations:** ^1^Unidad Móvil de Emergencias, Gerencia de Emergencias Sanitarias, Gerencia Regional de Salud de Castilla y León (SACYL), Spain; ^2^Centro de Simulación Clínica Avanzada, Departamento de Medicina, Dermatología y Toxicología, Universidad de Valladolid, Spain; ^3^Servicio de Urgencias, Complejo Asistencial Universitario de Palencia, Gerencia Regional de Salud de Castilla y León (SACYL), Spain; ^4^Unidad de Análisis de Datos (UAD) del Instituto de Investigación Sanitaria del Hospital de la Princesa (IIS-IP), Madrid, Spain; ^5^Gerencia de Atención Primaria de Salamanca, Gerencia Regional de Salud de Castilla y León (SACYL), Spain; ^6^Servicio de Urgencias, Hospital Universitario Rio Hortega de Valladolid, Gerencia Regional de Salud de Castilla y León (SACYL), Spain; ^7^Faculty of Health Sciences, Universidad de Castilla la Mancha, Talavera de la Reina, Spain

## Abstract

**Background:**

The aim of this study was to assess the role of prehospital point-of-care *N*-terminal probrain natriuretic peptide to predict sepsis, septic shock, or in-hospital sepsis-related mortality.

**Methods:**

A prospective, emergency medical service-delivered, prognostic, cohort study of adults evacuated by ambulance and admitted to emergency department between January 2020 and May 2021. The discriminative power of the predictive variable was assessed through a prediction model trained using the derivation cohort and evaluated by the area under the curve of the receiver operating characteristic on the validation cohort.

**Results:**

A total of 1,360 patients were enrolled with medical disease in the study. The occurrence of sepsis, septic shock, and in-hospital sepsis-related mortality was 6.4% (67 cases), 4.2% (44 cases), and 6.1% (64 cases). Prehospital National Early Warning Score 2 had superior predictive validity than quick Sequential Organ Failure Assessment and *N*-terminal probrain natriuretic peptide for detecting sepsis and septic shock, but *N*-terminal probrain natriuretic peptide outperformed both scores in in-hospital sepsis-related mortality estimation. Application of *N*-terminal probrain natriuretic peptide to subgroups of the other two scores improved the identification of sepsis, septic shock, and sepsis-related mortality in the group of patients with low-risk scoring.

**Conclusions:**

The incorporation of *N*-terminal probrain natriuretic peptide in prehospital care combined with already existing scores could improve the identification of sepsis, septic shock, and sepsis-related mortality.

## 1. Background

Detection and quick response by the emergency medical services (EMS) of time-dependent illness can make a big difference in the patient's condition [1]. Certain medical emergencies present a marked clinical manifestation, e.g., trauma, myocardial infarction, or stroke; all of them have specific codes and action guidelines already implemented to help in the management of these pathophysiological conditions [2] [3] [4]. However, other syndromic conditions such as sepsis present a more diffuse manifestation that may sometimes be unnoticed in its initial stages [5] [6].

The Third International Consensus Definitions for Sepsis and Septic Shock (Sepsis-3) [7] [8] and the International Guidelines for Management of Sepsis and Septic Shock 2021 “Surviving Sepsis Campaign” [9] [10] established the identification strategies and sequential lines of care to be followed in such cases. Hospital-based strategies have been developed for the early screening of sepsis, both in the intensive care units (ICU), such as the Sequential Organ Failure Assessment (SOFA) scores [11], and out of the ICU, such as the quick Sequential Organ Failure Assessment (qSOFA) or the National Early Warning Score 2 (NEWS2) [12] [13]. Additionally, other strategies based on the use of biomarkers such as lactate [14], C-reactive protein or procalcitonin [15], and adrenomedullin [16], the application of end-tidal carbon dioxide [17] or phenotyping [18] has been also proposed. In this sense, efforts to detect bedside sepsis by the EMS personnel are based on similar strategies to the ones adopted in the hospital setting, i.e., early warning scores, point-of-care (POC) testing, and specific training, but adapted to prehospital care [19]. However, the early identification of sepsis is still a challenge for EMS.

There is considerable interest in assessing new ways of early identification of sepsis with the *N*-terminal probrain natriuretic peptide (NT-proBNP), which is one of the most promising candidates [20]. As a result of the ongoing development of portable, robust, and reliable POC testing, it is now possible to obtain multiple biomarkers at bedside, clearly assisting the EMS personnel in the decision-making process since the very beginning of prehospital care [21].

Sepsis and septic shocks present a variable degree of multiorgan dysfunction syndromes that, in many cases, are associated to myocardial failure and with a significant rate of ICU-admissions and mortality [22, 23]. Determination of NT-proBNP is usually used to evaluate patients at risk of cardiovascular disorders, heart failure in particular, although it is also of prognostic utility in acute myocardial infarction or atrial fibrillation [24]. However, the evidence of its use in the diagnosis and stratification of sepsis is rather limited [25–28], or sparse in the prehospital scenario.

The primary endpoint of this study is aimed at determining the performance of prehospital point-of-care NT-proBNP to predict sepsis, septic shock, or in-hospital sepsis-related mortality (hereafter, sepsis-related mortality includes mortality by sepsis and septic shock) and to compare its performance with qSOFA or NEWS2-gold standards in nonICU settings-scores. Secondly, we checked whether the combination of NT-proBNP with qSOFA and NEWS2 can improve their prognostic performance in suspected patients of sepsis.

## 2. Methods

### 2.1. Study Set-Up and Ethical Issues

The present work is a prospective, ongoing, EMS-delivered, prognostic, cohort study of adults (>18 years old) evacuated by ambulance and admitted to emergency department (ED) between January 2020 and May 2021.

The study was carried out in the province of Valladolid (Spain). All calls for medical emergency help were delivered by an advanced life support (ALS) team, composed of two emergency medical technicians (EMT), an emergency registered nurse (ERN), and a physician. Once patients have been checked, cases requiring transfer were referred to the ED of the two tertiary university hospitals of the Public Health System, either in ALS or in one of the fourteen basic life support (BLS) units available in the area.

The institutional review board at the Hospital Universitario Rio Hortega and Hospital Clínico Universitario of Valladolid (reference: PI-049-19 and PI-GR-19-1258) approved the study protocol which was conducted in accordance with the Declaration of Helsinki. The study protocol was registered in the World Health Organization's International Clinical Trials Registry Platform (ICTRP) (https://doi.org/10.1186/ISRCTN48326533), and we followed the STrengthening the Reporting of OBservational studies in Epidemiology (STROBE) [29] statement.

### 2.2. Population

We screened all consecutive calls for medical emergency help (1-1-2 number) received during the trial period that were dispatched by the ALS unit and finally requiring high-priority transfer to the ED (either in ALS or BLS). The study included adult patients (>18 years old) with nontraumatic disease.

The following cases were excluded from the study: patients with traumatic diseases or poisoning (deliberate self-harm is classified under overdose or trauma, as appropriate), cases of cardiorespiratory arrest, pregnant women, end-of-life care situations, impossibility of conducting an analytical test through a venous blood sample, patients discharged in situ (after evaluation by the ALS physician), risky situations on the scene, and patients with no informed consent. Informed consent collection details can be found in supplementary methods.

### 2.3. Outcomes

The outcomes included sepsis, septic shock, and in-hospital sepsis-related mortality. For clinical operationalization, in accordance with the Third International Consensus Definitions for Sepsis and Septic Shock (Sepsis-3) [7] [30], an associate investigator reviewed the patient's electronic medical record (EMR) and collected data on sepsis (syndrome of multiorgan dysfunction with an increase of two or more SOFA score points), septic shock (deep syndrome of multiorgan dysfunction with the need for vasoactive drugs to maintain mean arterial pressure above 65 mmHg and lactate values above 2 mmol/L, after adequate fluid resuscitation), and in-hospital sepsis-related mortality.

Special attention was dedicated to congestive heart failure (CHF) as a comorbidity. A patient was considered suffering from CHF when the diagnosis was made by a specialist and recorded on the hospital's electronic medical record according to current heart failure guidelines [31, 32] and categorized into a subcohort for further analysis.

All cases of sepsis, septic shock, and in-hospital sepsis-related mortality were rechecked by the principal investigator. From now on, the terms mortality or in-hospital sepsis-related mortality will be indistinctly used.

### 2.4. Data Collection

All epidemiologic-demographic (age, sex, rural or urban area, intervention times, and type of ambulance) and clinical variables used to calculate qSOFA and/or NEWS2 (respiratory rate, oxygen saturation, supplemental oxygen use, systolic blood pressure, heart rate, temperature, and level of consciousness) were collected by the ERN at the scene or *en route*.

To determine NT-proBNP values, a trained ERN performed bedside POC by using a cobas h 232 analyzer (Roche Diagnostics, Mannheim, Germany) [21, 33, 34] in all of the patients included in the study. The age-adjusted Charlson comorbidity index (ACCI) was used to study comorbidities [35, 36].

Respiratory rate, systolic blood pressure, and level of consciousness were used to determine the qSOFA [37]. In addition, oxygen saturation, heart rate, temperature, and supplemental oxygen use are included in the NEWS estimate [38]. Two or more points on the qSOFA, or 5 or more points on the NEWS and possible infection, suggest the possibility of sepsis, and additional diagnostic steps are recommended to check the suspected diagnosis [39, 40].

Furthers details regarding data collection can be found in supplementary methods.

### 2.5. Statistical Analysis

Absolute values and percentages were used for categorical variables and median and interquartile ranges (IQR) for continuous variables because they did not follow a normal distribution. The characterization of the total sample and the association between each independent variable and the outcome were assessed by the Mann–Whitney *U* test or chi-squared test, when necessary.

Two main analyses were performed to answer the work's objectives. For the first objective, the discriminatory validity of the scores and the NT-proBNP was assessed by the area under the receiver operating characteristic (ROC) curve (AUC); specificity, sensitivity, positive predictive value, negative predictive value, positive likelihood ratio, and negative likelihood ratio of the scores and NT-proBNP were also calculated. Moreover, to compare performances, a Delong's test for AUCs comparisons and a decision curve analysis were used. To fulfill the second goal, a subset of patients was selected according to their qSOFA or NEWS scores using the following criteria: (i) patients with qSOFA scores inferior or equal to 1 and patients with scores superior or equal to 2, and (ii) for NEWS, scores superior or equal to 4 and NEWS scores superior or equal to 5. In this last case, only the AUC of the ROC was determined. The criteria for such scores' thresholds were based on the recommendations on the sepsis handling [7] and on the clinical use of NEWS2 [12].

All AUCs described in the work, except those from the second objective due to sample size, were determined from a validation cohort; i.e., two-thirds of the sample were used to fit the model and the other third to determine the validation capacity.

## 3. Results

### 3.1. Patient Baseline

**Figure 1:**
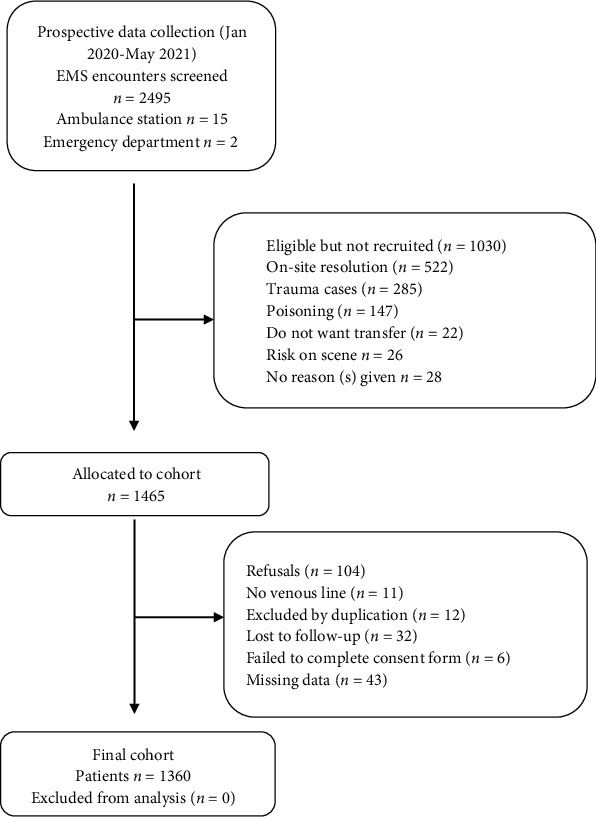
Participant inclusion flow diagram.

A total of 1,360 patients with medical disease meting the inclusion criteria were enrolled in the study (see Figure 1). Patients were predominantly elderly (median age 73 years, IQR: 59-83, range 18-99) with a considerably higher ratio of males; 780 (57.4%) were male and 580 (42.6%) were female. The overall inpatient ratio was 67.1%. The occurrence of sepsis, septic shock, and in-hospital sepsis-related mortalities was 6.4% (67 cases), 4.2% (44 cases), and 6.1% (64 cases), respectively. In cases of sepsis, the ICU-admission rate was 32.4% (36 cases), standing out the use of norepinephrine (36.9%, 41 cases) and the need for mechanical ventilation (25.2%, 28 cases). Patient characteristics are described in Table 1.

The sepsis origin was distributed as follows: respiratory (21 cases, 31.3%), abdominal (9 cases, 13.4%), urinary (21 cases, 31.3%), central nervous system (1 cases, 1.5%), skin (4 cases, 6%), multifactorial (7 cases, 10.4%), other (1 case, 1.5%), and with unclear origin (3 cases, 4.5). The septic shock origin was distributed as follows: respiratory (14 cases, 31.8%), abdominal (6 cases, 13.6%), urinary (9 cases, 20.5%), central nervous system (3 cases, 6.8%), skin (4 cases, 9.1%), multifactorial (3 cases, 6.8%), and with unclear origin (5 cases, 11.4%).

### 3.2. NT-proBNP Accuracy and Comparison to Scores

**Figure 2:**
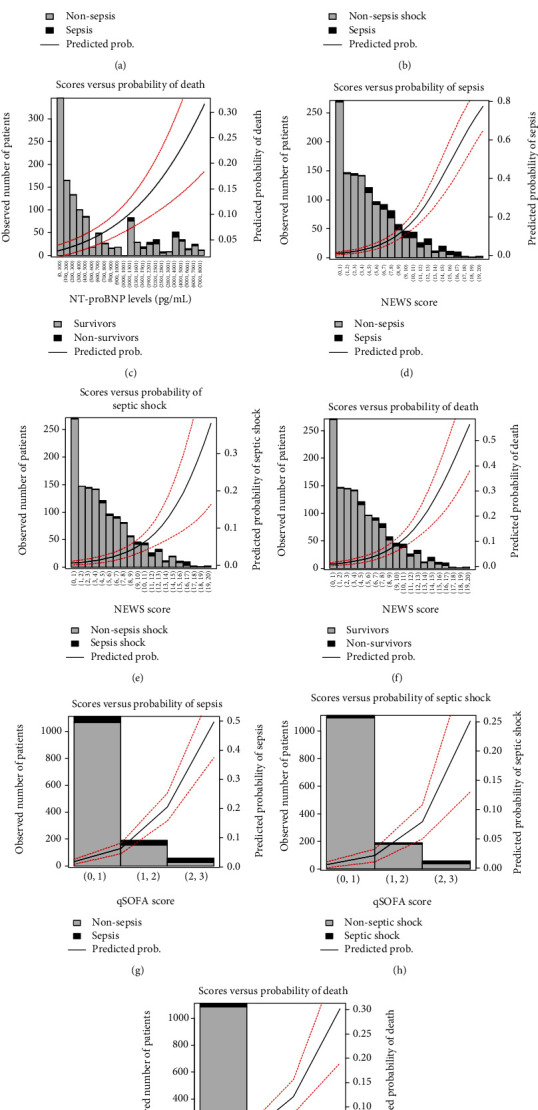
Observed number of cases for each of the outcomes: (a) sepsis, (b) septic shock, and (c) mortality for NT-proBNP; (d) sepsis, (e) septic shock, and (f) mortality for NEWS; and (g) sepsis, (h) septic shock, and (i) mortality for qSOFA. The grey shadowed area shows the predicted probability of the outcome.

The predictive validities of NT-proBNP, NEWS, and qSOFA for sepsis reached the following AUCs: 0.745 (95% CI: 0.671-0.819), 0.853 (95% CI: 0.802-0.904), and 0.859 (95% CI: 0.808-0.909), respectively. The same analysis was performed for septic shock reaching 0.807 (95% CI: 0.729-0.886), 0.843 (95% CI: 0.7391-0.946), and 0.822 (95% CI: 0.681-0.963), respectively and for in-hospital sepsis-related mortality reaching 0.860 (95% CI: 0.818-0.901), 0.845 (95% CI: 0.772-0.916), and 0.859 (95% CI: 0.787-0.932), respectively. Further parameters of the predictive validity can be found in supplementary eTable 1. Additionally, the observed number of cases for each outcome accordingly with the scores and the NT-proBNP is shown in Figure 2, which also shows the predicted probability of each outcome according to the value of the scores or the NT-proBNP levels. To determine the differences between the predictive validity of the NT-proBNP and the other two scores, Delong's test was used showing that the NT-proBNP presented a statistically significant lower AUC as compared to the other two scores (p <0.02 vs. NEWS; p <0.01 vs. qSOFA) in the case of sepsis. On the contrary, no statistical differences were found in the cases of septic shock (*p* < 0.622 vs. NEWS; *p* < 0.878 vs. qSOFA, both for septic shock) or mortality (*p* < 0.673 vs. NEWS; *p* < 0.989 vs. qSOFA, both corresponding for in-hospital sepsis-related mortality). Supplementary Figure 2 shows the AUC comparison for each outcome and the decision curve analysis for the comparison between NT-proBNP and the other two scores, for each of the 3 outcomes.

To evaluate the potential role of CHF on the scores and NT-proBNP, we compared the predictive validities of NT-proBNP, NEWS, and qSOFA for the cohort of patients with and without CHF. NEWS and qSOFA did not presented statistical difference between patients with and without CHF. However, NT-proBNP presented a lower AUC for the group with CHF as compared to the group without CHF, but this was only statistically significant for the case of mortality (Supplementary eTable3).

### 3.3. NT-proBNP Added Value to NEWS and qSOFA

The second objective of this works is aimed at determining the added value of NT-proBNP on the other two scores. Only patients without CHF were used in this case.  Table 2 shows the subgroup characteristics resulted from the categorization of patients according to NEWS or qSOFA.  Table 3 shows the predictive validity of NT-proBNP for each subgroup. As can be observed in Table 3, the NT-proBNP inclusion in different subgroups of NEWS2 and qSOFA improved the scores' predictive validity for sepsis, septic shock, and in-hospital sepsis-related mortality. Importantly, this improvement was greater for those subgroups of low risk (NEWS2 < 5 and qSOFA ≤ 1) in both scores. Further parameters of the predictive validity can be found in supplementary eTable 4.

## 4. Discussion

To our knowledge, this is the first study conducted in the prehospital care exploring the association between NT-proBNP and sepsis. We found that the prehospital NEWS2 score had superior specificity and sensitivity than qSOFA and NT-proBNP for detecting sepsis and septic shock, although the NT-proBNP was better to determine patients' in-hospital sepsis-related mortality as compared to qSOFA and NEWS2.

The implementation of scoring systems in prehospital care is a clinical reality [41, 42]. Their simplicity, the use of commonly vital signs, their elevated discriminative capacity, the easy interpretation, and their fast-learning curve make the score optimal tools for bedside use [43]. Probably the most widely implemented scoring system at an international scale is the NEWS, developed by the Royal College of Physicians of London [44], with its latest update NEWS2 [12], bearing a recognized efficacy in several clinical conditions, including sepsis [38, 45]. The most widely accepted early identification score for outside-hospital of sepsis is the qSOFA, proposed in the Third International Consensus Definitions for Sepsis and Septic Shock (Sepsis-3) [7] [46].

The work presented here showed that both qSOFA and NEWS2 scores have a good forecasting capacity for the classification of sepsis, septic shock, and in-hospital sepsis-related mortality, confirming them as fundamental tools for the diagnosis of sepsis outside the hospital settings [47, 48]. Our findings are in accordance with similar investigations, albeit with superior statistical performance. Silcock et al. [49] tested qSOFA vs. NEWS in the prehospital setting with AUCs of 0.67 and 0.74, respectively, although on unselected patients. Liu et al. [50] analyzed the predictive ability of qSOFA and NEWS in ICU-admission and in-hospital mortality in patients with suspected infection showing, an AUC of 0.78 and 0.87, respectively; Mellhammar et al. [51] obtained similar results. Nieves Ortega et al. [52] demonstrated the superior performance of NEWS over qSOFA, with an AUC of 0.85 vs. 0.79. In summary, qSOFA yielded high specificity, but low sensitivity. NEWS2 outperformed the predictive ability for sepsis-related outcomes, findings that agree with our own results [39, 53, 54].

On the other hand, the technical possibility of bedside POC testing has encouraged EMS to implement in their guidelines the use of biomarkers in prehospital care to improve both the diagnostic and prognostic capabilities. Research concerning the clinical usefulness of prehospital NT-proBNP is scarce [55, 56], finding more evidence about its interpretation in patients with sepsis and especially with septic shock in the hospital setting [25, 57]. Sepsis-associated myocardial depression is a mayor expression of multiorgan dysfunction in the septic patient [58]. It is well-known that NT-proBNP (secreted by the ventricles in response to distension or increased ischemia on demand) has a potent vasodilatory effect, inhibiting the physiological renin-angiotensin-aldosterone and the sympathetic nervous systems [26]. Recent evidence supports the suggestion that myocardial failure (and especially right ventricular dysfunction) is associated with increased short-term mortality in sepsis and septic shock [59]. About 25% of patients with sepsis and one-half of patients with septic shock present myocardial involvement, including biventricular thickening, diminished contractility, and diastolic dysfunction [60]. Currently, the best available treatment for myocardial dysfunction in sepsis is appropriate volume-targeted resuscitation, followed by the addition of inotropes, to ensure sufficient perfusion pressure for metabolic requirements [61–63].

As a single biomarker, NT-proBNP did not improved the ability to discriminate sepsis or septic shock as compared to NEWS2 or qSOFA, nor its net benefit. Despite the fact that NT-proBNP provides encouraging performances compared to specific in-hospital biomarkers to manage sepsis, like lactate, C-reactive protein, or procalcitonin [15, 64, 65], it did not improve bottom-line results [66]. More important, this biomarker can identify patients with sepsis and in septic shock and predict sepsis-related mortality with high reliability in the group of patients with low-risk scoring (NEWS2 < 5 and qSOFA ≤ 1) [67]. The analysis of subgroups with CHF suggested that the interpretation of NT-proBNP should be handled with prudence. However, in CHF-free patients, the use of this biomarker, especially in low-risk patients (NEWS2 < 5 and qSOFA ≤ 1), may be helpful to identify suspected sepsis patients with an uncertain clinical course.

Patients with sepsis and septic shock have unacceptably dramatic mortality rates [68]. Despite the efforts shown by different organizations [9] [69], mortality rates remain extremely high today [70]. Sepsis is featured by fuzzy symptomatology and limited clinical manifestations in the initial stages, making its rapid detection a challenge for the EMS personnel [71]. Late recognition frequently implies that its diagnosis is often accompanied with syndromes of multiorgan dysfunction already established, delaying the therapeutic measures [72, 73].

## 5. Limitations

This study has several limitations. First, it is a convenience cohort, in a single province with a relatively small number of events. To minimize bias, we included 24/7 recruitments, urban and rural backgrounds, BLS or ALS transfers, for the duration of the follow-up; and as an add-on, validation was performed in different cohorts to check the consistency of the NT-proBNP and the scores employed. Second, the proportion of older adults is significantly elevated but does not exceed the ones of previous epidemiological studies and is in line with the general increase of elderly worldwide, especially in our neighboring countries. Third, the data extractors were not blinded. To avoid bias, the criteria of sepsis, septic shock, and in-hospital sepsis-related mortality were based on the Third International Consensus Definitions for Sepsis and Septic Shock (Sepsis-3) [7]. A physician from each hospital collected all hospital variables, and in case of sepsis-related outcomes, the event was double-checked by the principal investigator. Fourth, although POC is clearly now being implemented in ambulances, these devices are not currently generalizable on all EMS-systems. Finally, the study began in January 2020 and stopped in May 2021, during the coronavirus 19 (COVID-19) pandemic. Broad epidemiological studies are needed to understand the impact of the ongoing pandemic on non-COVID-19 disease and identify there has been underdiagnosis of sepsis or unexpected excess mortality during this period.

## 6. Conclusion

NT-proBNP was a strong predictor of in-hospital sepsis-related mortality, similar to the other two scores, but not for recognizing sepsis and septic shock, in which NEWS2 was better. Moreover, the NT-proBNP add-on to the other two scores improved sepsis prediction in patients at low risk of sepsis. Therefore, complementing scoring systems with POC should be a must in prehospital clinical practice since starting life support as quickly as possible is key to improve survival and minimize complications.

## Figures and Tables

**Table 1:** Baseline patients' characteristic-based sepsis diagnosis.

	Total	Sepsis	Nonsepsis	*p* value^b^
No. (%) with data^a^	1360	111 (8.2)	1249 (91.8)	
Age (y)	73 (59-83)	77 (65-87)	73 (59-82)	.011
Sex (female)	580 (42.6)	40 (36)	540 (43.2)	.142
Time (minutes)				
Arrival	11 (9-15)	12 (9-17)	11 (9-15)	.282
Assistance	33 (27-41)	34 (28-42)	33 (26-41)	.025
Transfer	11 (8-16)	12 (10-17)	11 (8-16)	.086
Advanced life support	888 (65.3)	73 (65.8)	434 (34.7)	.913
Zone (urban)	1056 (77.6)	80 (72.1)	976 (78.1)	.141
Prehospital care				
Respiratory rate (bpm)	18 (14-26)	27 (23-33)	18 (14-24)	<.001
Pulse oximetry saturation (%)	96 (92-98)	92 (82-95)	96 (93-98)	<.001
Supplemental oxygen	150 (11)	36 (32.4)	114 (9.1)	<.001
Systolic arterial pressure (mmHg)	137 (116-158)	106 (88-135)	139 (120-160)	<.001
Heart rate (bpm)	85 (70-105)	104 (83-124)	84 (70-104)	<.001
Temperature (°C)	36.1 (35.8-36.7)	37 (36-38.6)	36.1 (35.8-36.6)	<.001
Glasgow coma scale (points)	15 (15-15)	14 (11-15)	15 (15-15)	<.001
Volume (mL)	250 (250-250)	500 (25-1000)	250 (250-250)	<.001
Mechanical ventilation	113 (8.3)	17 (15.3)	96 (7.7)	.005
Norepinephrine	52 (3.8)	13 (11.7)	39 (3.1)	<.001
qSOFA (points)	1 (0-1)	2 (1-3)	0 (0-1)	<.001
NEWS2 (points)	4 (2-8)	10 (7-13)	4 (2-7)	<.001
NT-proBNP (pg/mL)	328 (98-1147)	1769 (609-3433)	300 (75-1044)	<.001
Hospital outcomes				
Hospital-inpatient	912 (67.1)	111 (100)	801 (64.1)	<.001
Hospitalization time (day)	4 (0-9)	8 (2-20)	4 (0-7)	<.001
Intensive care unit-admission	139 (10.2)	36 (32.4)	103 (8.2)	<.001
Mechanical ventilation	131 (9.6)	28 (25.2)	103 (8.2)	<.001
Norepinephrine	121 (8.9)	41 (36.9)	80 (6.4)	<.001
Septic shock	44 (3.2)	44 (39.6)	0	NA
In-hospital mortality	64 (4.7)	64 (57.7)	0	NA
ACCI (points)	5 (3-7)	7 (6-10)	5 (3-7)	<.001
AIDS	20 (1.5)	2 (1.9)	18 (1.4)	.762
Solid tumor metastatic	65 (4.8)	11 (9.9)	54 (4.3)	.008
Liver disease severe	65 (4.8)	11 (9.9)	54 (4.3)	.008
Lymphoma	15 (1.1)	3 (2.7)	12 (1)	.092
Leukemia	18 (1.3)	2 (1.8)	16 (1.3)	.646
Solid tumor localized	314 (23.1)	39 (35.1)	275 (22)	.002
DM end organ damage	204 (15)	13 (11.7)	191 (15.3)	.312
Severe chronic kidney disease	195 (14.3)	25 (22.5)	170 (13.6)	.010
Hemiplegia	102 (7.5)	18 (16.2)	84 (6.7)	<.001
DM uncomplicated	204 (15)	13 (811.7)	191 (15.3)	.312
Liver disease mild	47 (3.5)	4 (3.6)	43 (3.4)	.929
Peptic ulcer disease	180 (13.2)	19 (17.1)	161 (12.9)	.208
Connective disease	125 (9.2)	15 (13.5)	110 (8.8)	.100
COPD	303 (22.3)	36 (32.4)	267 (21.4)	.007
Dementia	210 (15.4)	36 (32.4)	174 (13.9)	<.001
Cerebrovascular disease	172 (12.6)	18 (16.2)	154 (12.3)	.238
Peripheral vascular disease	198 (14.6)	16 (814.4)	182 (14.6)	.964
Congestive heart failure	320 (23.5)	37 (33.3)	283 (22.7)	.011
Myocardial infarction	333 (24.5)	21 (18.9)	312 (25)	.147

qSOFA: quick Sequential Organ Failure Assessment; NEWS2: National Early Warning Score 2; NT-proBNP: *N*-terminal probrain natriuretic peptide; NA: not applicable; ACCI: age-adjusted Charlson comorbidity index; AIDS: acquired immunodeficiency syndrome; DM: diabetes mellitus; COPD: chronic obstructive pulmonary disease; PVD: peripheral vascular disease. ^a^Values expressed as total number (fraction) and medians [25 percentile-75 percentile], as appropriate. ^b^The Mann–Whitney *U* test, *t*-test, or chi-squared test was used as appropriate.

**Table 2:** Prehospital sepsis prediction using early warning scores.

	Low risk	High risk	
	Quick Sequential Organ Failure Assessment		
	0-1 points	2-3 points	*p* value^b^
No. (%) with data^a^	1112 (81.8)	248 (18.2)	
Age (y)	73 (59-82)	76 (61-85)	.275
Sex (female)	487 (43.4)	93 (37.5)	.017
Prehospital care			
Volume (mL)	250 (250-250)	500 (250-500)	<.001
Mechanical ventilation	51 (4.6)	62 (25)	<.001
Norepinephrine	10 (0.9)	42 (16.9)	<.001
NT-proBNP (pg/mL)	289 (67-1038)	696 (224-2633)	<.001
Hospital outcomes			
ACCI (points)	5 (3-7)	7 (5-9)	<.001
Hospital-inpatient	695 (62.5)	217 (87.5)	<.001
Hospitalization time (day)	4 (0-8)	5 (1-11)	.008
Intensive care unit-admission	83 (7.5)	56 (22.6)	<.001
Mechanical ventilation	74 (6.7)	57 (23)	<.001
Norepinephrine	53 (4.8)	68 (27.4)	<.001
Sepsis	44 (4)	67 (27)	<.001
Septic shock	16 (1.4)	28 (11.3)	<.001
In-hospital mortality	25 (2.2)	39 (15.7)	<.001
	National Early Warning Score 2		
	≤4 points	≥5 points	*p* value^b^
No. (%) with data^a^	705 (51.8)	655 (48.2)	
Age (y)	71 (56-80)	75 (64-85)	<.001
Sex (female)	283 (40.1)	297 (45.3)	.053
Prehospital care			
Volume (mL)	250 (250-250)	250 (250-500)	<.001
Mechanical ventilation	9 (1.3)	104 (15.9)	<.001
Norepinephrine	0	52 (7.9)	<.001
NT-proBNP (pg/mL)	208 (0-559)	650 (201-2399)	<.001
Hospital outcomes			
ACCI (points)	5 (2-7)	6 (4-9)	<.001
Hospital-inpatient	381 (54)	531 (81.1)	<.001
Hospitalization time (day)	3 (0-7)	6 (1-11)	<.001
Intensive care unit-admission	31 (4.4)	108 (16.5)	<.001
Mechanical ventilation	26 (3.7)	105 (16)	<.001
Norepinephrine	13 (1.8)	108 (16.5)	<.001
Sepsis	8 (1.1)	103 (15.7)	<.001
Septic shock	4 (0.6)	40 (6.1)	<.001
In-hospital mortality	4 (0.6)	60 (9.2)	<.001

NT-proBNP: *N*-terminal probrain natriuretic peptide; ACCI: age-adjusted Charlson comorbidity index; NA: not applicable. ^a^Values expressed as total number (fraction) and medians [25 percentile-75 percentile], as appropriate. ^b^The Mann–Whitney *U* test, *t*-test, or chi-squared test was used as appropriate.

**Table 3:** Predictive validity of NT-proBNP according to NEWS and qSOFA subgroups.

Sepsis	AUC (95% CI)
NEWS < 5	0.880 (0.757-1)
NEWS ≥ 5	0.713 (0.654-0.773)
qSOFA ≤ 1	0.809 (0.735-0.881)
qSOFA > 1	0.705 (0.621-0.788)
Septic shock	
NEWS < 5	0.862 (0.643-1)
NEWS ≥ 5	0.768 (0.695-0.841)
qSOFA ≤ 1	0.908 (0.839-0.976)
qSOFA > 1	0.708 (0.605-0.812)
Mortality	
NEWS < 5	0.940 (0.874-1)
NEWS ≥ 5	0.828 (0.772-0.885)
qSOFA ≤ 1	0.903 (0.855-0.950)
qSOFA > 1	0.823 (0.742-0.904)

AUC: area under the curve; 95% CI: 95% confidence interval. ^a^The low number of cases do not allow the validation procedure.

## Data Availability

The datasets analyzed during the current study are available from the corresponding author on reasonable request.

## Additional Points

*Presentations*. This study was not previously presented in conferences.

## Supplementary Materials

Supplementary Materials The supplementary material contains the following: Supplementary eTable 1: predictive validity of NT-proBNP according to NEWS and qSOFA subgroups. Supplementary figure 2: AUC comparison for each outcome (a) sepsis, (b) septic shock, and (c) mortality for NT-proBNP (red line), NEWS (green line), and qSOFA (blue line); and the decision curve analysis for the comparison between NT-proBNP and NEWS and qSOFA for (d) sepsis, (e) septic shock, and (f) mortality. Supplementary eTable 3: predictive validity comparison of NT-proBNP, NEWS, and qSOFA for the cohort of patients with and without CHF. Supplementary eTable 4: predictive validity of NT-proBNP according to NEWS and qSOFA.
