# Supplementary material for: Combination of Prehospital NT-proBNP with qSOFA and NEWS to Predict Sepsis and Sepsis-Related Mortality
Source: Dis Markers. 2022 Feb 23;2022:5351137. doi: 10.1155/2022/5351137 (PMC8886755; doi:10.1155/2022/5351137)
Supplement: Supplementary Materials — The supplementary material contains the following: Supplementary eTable 1: predictive validity of NT-proBNP according to NEWS and qSOFA subgroups. Supplementary figure 2: AUC comparison for each outcome (a) sepsis, (b) septic shock, and (c) mortality for NT-proBNP (red line), NEWS (green line), and qSOFA (blue line); and the decision curve analysis for the comparison between NT-proBNP and NEWS and qSOFA for (d) sepsis, (e) septic shock, and (f) mortality. Supplementary eTable 3: predictive validity comparison of NT-proBNP, NEWS, and qSOFA for the cohort of patients with and without CHF. Supplementary eTable 4: predictive validity of NT-proBNP according to NEWS and qSOFA. [file 5351137.f1.zip › supplementary eTable1.docx]

| **Sepsis** | **AUC (95%CI)** | **Youden index Specificity (optimal cutoff)^a^** | **Youden index Sensitivity (optimal cutoff)^a^** | **Global Specificity (95%CI)^b^** | **Global Sensitivity (95%CI)^b^** | **Positive predictive value (95%CI)** | **Negative predictive value (95%CI)** | **Positive likelihood ratio (95%CI)** | **Negative likelihood ratio (95%CI)** |
| --- | --- | --- | --- | --- | --- | --- | --- | --- | --- |
| NT-proBNP | 0.745 (95% CI: 0.671 - 0.819) | 61.56 (443) | 80.18 (443) | 87.46 (87.19-87.74) | 28.78 (28.31-29.25) | 18.61(18.55-18.66) | 93.50 (93.47-93.53) | 2.59 (2.58-2.60) | 0.79 (0.78-0.79) |
| NEWS | 0.853 (95% CI: 0.802 - 0.904) | 72.13 (6.5) | 80.18 (6.5) | 72.53 (57.59-87.47) | 52.60 (35.26-69.93) | 22.25 (15.11-29.38) | 95.49 (94.16-96.81) | 3.84 (2.34-5.34) | 0.54 (0.38-0.70) |
| qSOFA | 0.859 (95% CI: 0.808 - 0.909) | 85.50 (1.5) | 60.36 (1.5) | 58.51 (0-100) | 69.82 (17.82-100) | 25.68 (0-57.69) | 96.11 (90.41-100) | 4.98 (0-13.67) | 0.46 (0-1.15) |
|  |  |  |  |  |  |  |  |  |  |
| **Septic shock** |  |  |  |  |  |  |  |  |  |
| NT-proBNP | 0.807 (95% CI: 0.729 - 0.886) | 66.94 (675) | 84.09 (675) | 86.89 (86.61-87.17) | 36.21 (35.71-36.70) | 9.98 (9.95-10.02) | 97.71 (97.70-97.73) | 3.33 (3.32-3.34) | 0.70 (0.70-0.71) |
| NEWS | 0.843 (95% CI: 0.739 - 0.946) | 82.14 (8.5) | 65.90 (8.5) | 71.30 (56.33-86.28) | 54.11 (37.61-70.61) | 10.97 (6.29-15.66) | 98.24 (97.76-98.73) | 4.21 (2.01-6.41) | 0.54 (0.39-0.69) |
| qSOFA | 0.822 (95% CI: 0.681 - 0.963) | 83.28 (1.5) | 63.63 (1.5) | 57.20 (0-100) | 73.86 (31.12-100) | 12.78 (0-32.88) | 98.65 (96.93-100) | 5.01 (0-14.17) | 0.41 (0-0.94) |
|  |  |  |  |  |  |  |  |  |  |
| **Mortality** |  |  |  |  |  |  |  |  |  |
| NT-proBNP | 0.860 (95% CI: 0.818 - 0.901) | 76.54 (1090.5) | 84.37 (1090.5) | 87.42 (87.14-87.70) | 39.81 (39.25-40.38) | 15.58 (15.53-15.63) | 96.90 (96.87-96.93) | 3.76 (3.74-3.77) | 0.65 (0.65-0.66) |
| NEWS | 0.845 (95% CI: 0.772 - 0.916) | 70.44 (6.5) | 84.37 (6.5) | 71.72 (56.76-86.68) | 54.61 (37.29-71.94) | 14.19 (9.32-19.06) | 97.54 (96.76-98.32) | 3.72 (2.28-5.17) | 0.52 (0.35-0.68) |
| qSOFA | 0.859 (95% CI: 0.787 - 0.932) | 83.87 (1.5) | 60.93 (1.5) | 57.54 (0-100) | 71.09 (21.02-100) | 15.79 (0-36.92) | 97.86 (94.62-100) | 4.31 (0-11.29) | 0.44 (0-1.13) |
|  |  |  |  |  |  |  |  |  |  |

Supplementary eTable 1. Predictive validity of NT-proBNP according to NEWS and qSOFA subgroups.

*Abbreviations* AUC: Area under the curve; 95%CI: 95% Confidence interval.

^a^Refers to the sensitivity and specificity which is found at the optimal cutoff (Youden index) value of each score. The value between parentheses represents the optimal cutoff score value.

^b^Refers to the mean sensitivity and specificity, which it is obtained by averaging the sensitivity and specificity throughout all values from each score.
