# Supplementary material for: Combination of Prehospital NT-proBNP with qSOFA and NEWS to Predict Sepsis and Sepsis-Related Mortality
Source: Dis Markers. 2022 Feb 23;2022:5351137. doi: 10.1155/2022/5351137 (PMC8886755; doi:10.1155/2022/5351137)
Supplement: Supplementary Materials — The supplementary material contains the following: Supplementary eTable 1: predictive validity of NT-proBNP according to NEWS and qSOFA subgroups. Supplementary figure 2: AUC comparison for each outcome (a) sepsis, (b) septic shock, and (c) mortality for NT-proBNP (red line), NEWS (green line), and qSOFA (blue line); and the decision curve analysis for the comparison between NT-proBNP and NEWS and qSOFA for (d) sepsis, (e) septic shock, and (f) mortality. Supplementary eTable 3: predictive validity comparison of NT-proBNP, NEWS, and qSOFA for the cohort of patients with and without CHF. Supplementary eTable 4: predictive validity of NT-proBNP according to NEWS and qSOFA. [file 5351137.f1.zip › supplementary eTable3.docx]

Supplementary eTable 3. Predictive validity comparison of NT-proBNP, NEWS, and qSOFA for the cohort of patients with and without CHF.

| **Sepsis** | **AUC patients with CHF** | **AUC patients without CHF** | **DeLong's test pvalue** |
| --- | --- | --- | --- |
| NT-proBNP | 0.607 | 0.789 | 0.08 |
| NEWS | 0.791 | 0.895 | 0.203 |
| qSOFA | 0.759 | 0.881 | 0.21 |
|  |  |  |  |
| **Septic shock** |  |  |  |
| NT-proBNP | 0.771 | 0.868 | 0.65 |
| NEWS | 0.751 | 0.882 | 0.37 |
| qSOFA | 0.698 | 0.837 | 0.59 |
|  |  |  |  |
| **Mortality** |  |  |  |
| NT-proBNP | 0.671 | 0.903 | 0.007* |
| NEWS | 0.907 | 0.825 | 0.24 |
| qSOFA | 0.852 | 0.759 | 0.35 |
|  |  |  |  |

*Abbreviations* AUC: Area under the curve

*Statistically significant differences.
