# Supplementary material for: Combination of Prehospital NT-proBNP with qSOFA and NEWS to Predict Sepsis and Sepsis-Related Mortality
Source: Dis Markers. 2022 Feb 23;2022:5351137. doi: 10.1155/2022/5351137 (PMC8886755; doi:10.1155/2022/5351137)
Supplement: Supplementary Materials — The supplementary material contains the following: Supplementary eTable 1: predictive validity of NT-proBNP according to NEWS and qSOFA subgroups. Supplementary figure 2: AUC comparison for each outcome (a) sepsis, (b) septic shock, and (c) mortality for NT-proBNP (red line), NEWS (green line), and qSOFA (blue line); and the decision curve analysis for the comparison between NT-proBNP and NEWS and qSOFA for (d) sepsis, (e) septic shock, and (f) mortality. Supplementary eTable 3: predictive validity comparison of NT-proBNP, NEWS, and qSOFA for the cohort of patients with and without CHF. Supplementary eTable 4: predictive validity of NT-proBNP according to NEWS and qSOFA. [file 5351137.f1.zip › supplementary eTable4.docx]

Supplementary eTable 4. Predictive validity of NT-proBNP according to NEWS and qSOFA

| **Sepsis** | **AUC (95%CI)** | **Youden index Specificity (optimal cutoff)^a^** | **Youden index Sensitivity (optimal cutoff)^a^** | **Specificity (95%CI)** | **Sensitivity (95%CI)** | **Positive predictive value (95%CI)** | **Negative predictive value (95%CI)** | **Positive likelihood ratio (95%CI)** | **Negative likelihood ratio (95%CI)** |
| --- | --- | --- | --- | --- | --- | --- | --- | --- | --- |
| NEWS <5 | 0.880 (0.757-1) | 87.47 (775.5) | 80 (775.5) | 95.21 (95.00-95.41) | 17.80 (17.22-18.37) | 2.01 (1.95-2.07) | 99.27 (99.27-99.28) | 2.46 (2.39-2.54) | 0.84 (0.83-0.85) |
| NEWS ≥5 | 0.713 (0.654-0.773) | 62.14 (443) | 75.36 (443) | 88.83 (88.54-89.11) | 20.47 (20.00-20.94) | 24.84 (24.75-24.93) | 86.80 (86.75-86.86) | 1.90 (1.89-1.91) | 0.87 (0.86-0.87) |
| qSOFA ≤1 | 0.809 (0.735-0.881) | 73.89 (446.5) | 78.57 (446.5) | 93.22 (92.99-93.45) | 22.00 (21.54-22.46) | 12.44 (12.38-12.49) | 97.36 (97.34-97.37) | 4.29 (4.26-4.31) | 0.81 (0.81-0.82) |
| qSOFA>1 | 0.705 (0.621-0.788) | 67.71 (549) | 69.56 (549) | 88.67 (88.38-88.97) | 19.25 (18.76-19.74) | 34.73 (34.59-34.86) | 76.05 (75.96-76.14) | 1.51 (1.51-1.52) | 0.88 (0.88-0.89) |
|  |  |  |  |  |  |  |  |  |  |
| **Septic shock** |  |  |  |  |  |  |  |  |  |
| NEWS <5 | 0.862 (0.643-1) | 63.95 (242) | 100 (242) | 95.18 (94.98-95.39) | 21.95 (21.31-22.59) | 1.78 (1.73-1.84) | 99.59 (99.58-99.59) | 3.64 (3.53-3.76) | 0.80 (0.79-0.81) |
| NEWS ≥5 | 0.768 (0.695-0.841) | 57.63 (422.5) | 89.28 (422.5) | 88.18 (87.88-88.48) | 24.08 (23.51-24.64) | 9.49 (9.39-9.60) | 94.96 (94.93-94.99) | 1.67 (1.65-1.69) | 0.82 (0.82-0.83) |
| qSOFA ≤1 | 0.908 (0.839-0.976) | 89.13 (1233.5) | 90.90 (1233.5) | 93.07 (92.84-93.30) | 33.56 (32.91-34.20) | 6.76 (6.70-6.83) | 99.13 (99.12-99.14) | 5.74 (5.68-5.79) | 0.69 (0.68-0.69) |
| qSOFA>1 | 0.708 (0.605-0.812) | 50.98 (340) | 90 (340) | 87.23 (86.92-87.55) | 18.54 (18.01-19.08) | 9.53 (9.34-9.72) | 89.58 (89.53-89.63) | 0.90 (0.88-0.92) | 0.90 (0.89-0.90) |
|  |  |  |  |  |  |  |  |  |  |
| **Mortality** |  |  |  |  |  |  |  |  |  |
| NEWS <5 | 0.940 (0.874-1) | 87.34 (775.5) | 100 (775.5) | 95.19 (94.99-95.40) | 23.94 (23.23-24.65) | 1.84 (1.78-1.89) | 99.60 (99.60-99.60) | 3.75 (3.63-3.87) | 0.77 (0.77-0.78) |
| NEWS ≥5 | 0.828 (0.772-0.885) | 78.11 (1090.5) | 80 (1090.5) | 89.03 (88.73-89.32) | 31.91 (31.34-32.49) | 22.94 (22.84-23.04) | 94.40 (94.36-94.44) | 3.68 (3.66-3.70) | 0.73 (0.72-0.73) |
| qSOFA ≤1 | 0.903 (0.855-0.950) | 73.62 (447.5) | 100 (447.5) | 93.17 (92.94-93.40) | 34.24 (33.70-34.78) | 11.53 (11.47-11.60) | 98.90 (98.89-98.91) | 8.04 (7.99-8.09) | 0.68 (0.67-0.69) |
| qSOFA>1 | 0.823 (0.742-0.904) | 74.49 (846) | 87.50 (846) | 89.16 (88.86-89.47) | 29.56 (28.94-30.18) | 32.24 (32.13-32.35) | 89.41 (89.32-89.49) | 3.01 (2.99-3.02) | 0.75 (0.74-0.75) |

*Abbreviations* AUC: Area under the curve.

^a^Refers to the sensitivity and specificity which is found at the optimal cutoff (Youden index) value of each score. The value between parentheses represents the optimal cutoff score value.

^b^Refers to the mean sensitivity and specificity, which it is obtained by averaging the sensitivity and specificity throughout all values from each score.

The low number of cases do not allow the validation procedure.
