# Supplementary material for: Combination of Prehospital NT-proBNP with qSOFA and NEWS to Predict Sepsis and Sepsis-Related Mortality
Source: Dis Markers. 2022 Feb 23;2022:5351137. doi: 10.1155/2022/5351137 (PMC8886755; doi:10.1155/2022/5351137)
Supplement: Supplementary Materials — The supplementary material contains the following: Supplementary eTable 1: predictive validity of NT-proBNP according to NEWS and qSOFA subgroups. Supplementary figure 2: AUC comparison for each outcome (a) sepsis, (b) septic shock, and (c) mortality for NT-proBNP (red line), NEWS (green line), and qSOFA (blue line); and the decision curve analysis for the comparison between NT-proBNP and NEWS and qSOFA for (d) sepsis, (e) septic shock, and (f) mortality. Supplementary eTable 3: predictive validity comparison of NT-proBNP, NEWS, and qSOFA for the cohort of patients with and without CHF. Supplementary eTable 4: predictive validity of NT-proBNP according to NEWS and qSOFA. [file 5351137.f1.zip › supplementary Figure2.pdf]

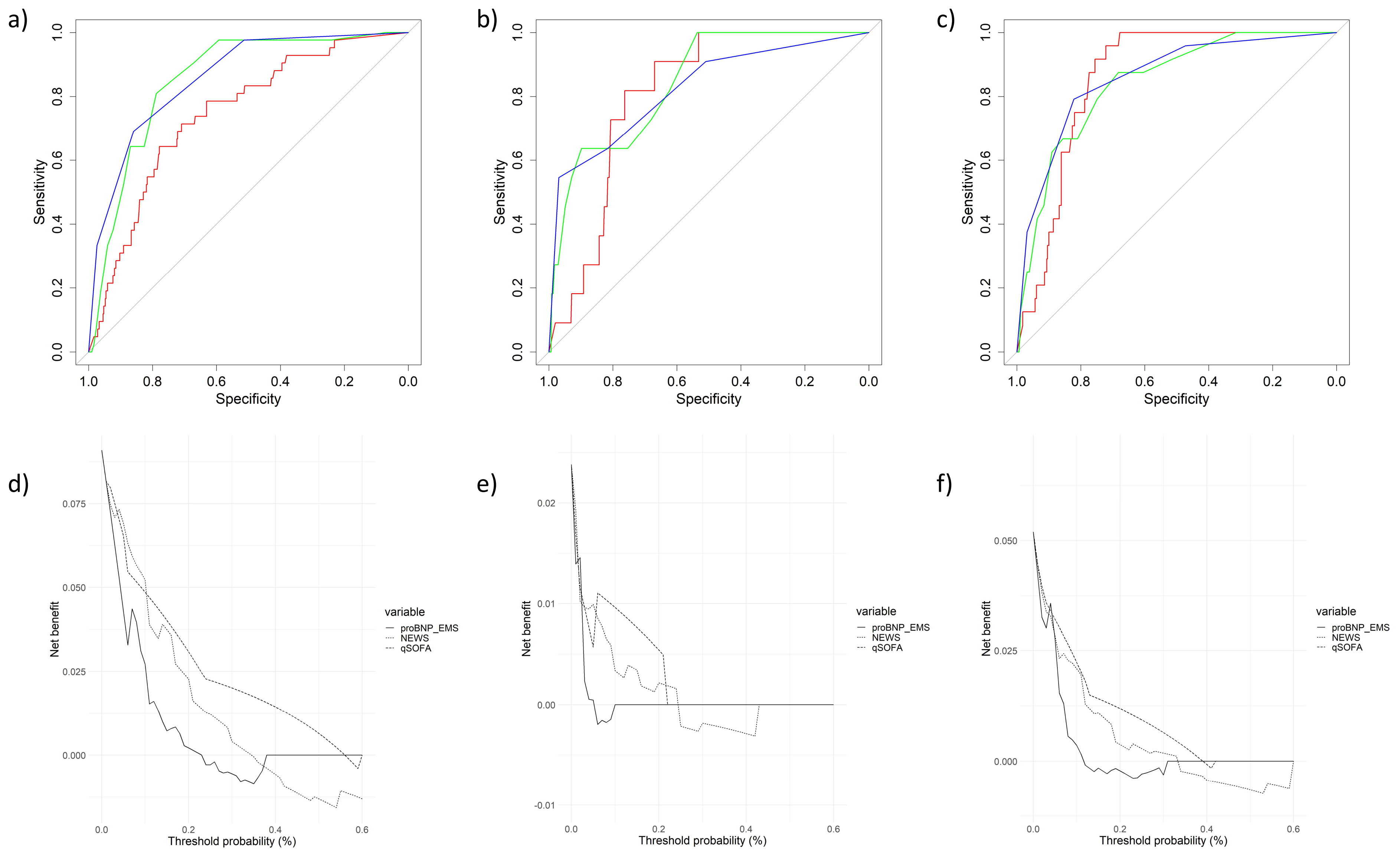

AUC comparison for each outcome (a) sepsis, (b) septic shock and (c) mortality for NT-proBNP (red line), NEWS (green line) and qSOFA (blue line); and the decision curve analysis for the comparison between NT-proBNP and NEWS and qSOFA for (d) sepsis, (e) septic shock and (f) mortality.
